# The Effect of Insomnia on the Outcomes of Physical Therapy in Patients with Cervical and Lumbar Pain in Clinical Practice

**DOI:** 10.3390/medicina60111873

**Published:** 2024-11-15

**Authors:** Milan Djordjic, Aleksandra Jurisic Skevin, Vesna Grbovic, Ermin Fetahovic, Sofija Colovic, Milan Zaric, Tatjana Boskovic Matic, Olivera Radmanovic, Vladimir Janjic

**Affiliations:** 1 Department of Communication Skills, Ethics, and Psychology, Faculty of Medical Sciences, University Kragujevac, 34000 Kragujevac, Serbia; mcpikac@gmail.com (M.D.); erminfetahovic96@gmail.com (E.F.); 2 Department of Physical Medicine and Rehabilitation, Faculty of Medical Sciences, University of Kragujevac, 34000 Kragujevac, Serbia; jsaleksandra@gmail.com (A.J.S.); grbovicvesna72@gmail.com (V.G.); 3 Department of Physical Medicine and Rehabilitation, University Clinical Center of Kragujevac, 34000 Kragujevac, Serbia; 4 Department of Psychiatry, Faculty of Medical Sciences, University of Kragujevac, 34000 Kragujevac, Serbia; sofi007@gmail.com (S.C.); vladadok@yahoo.com (V.J.); 5 Psychiatry Clinic, University Clinical Center Kragujevac, 34000 Kragujevac, Serbia; 6 Department of Biochemistry, Faculty of Medical Sciences, University of Kragujevac, 34000 Kragujevac, Serbia; zaricmilan@gmail.com; 7 Department of Neurology, Faculty of Medical Sciences, University of Kragujevac, 34000 Kragujevac, Serbia; 8 Clinic for Rheumatology and Allergology, University Clinical Center Kragujevac, 34000 Kragujevac, Serbia; oljaradmanovic@gmail.com; 9 Faculty of Medical Sciences, University of Kragujevac, 34000 Kragujevac, Serbia

**Keywords:** pain, insomnia, SF-MPQ, NSE—neuron-specific enolase, NEF—neurofilament polypeptide, protein S100B

## Abstract

*Background and Objectives*: The objective of the study is to determine whether there is a difference in physical therapy outcomes in patients with cervical and/or lumbar pain who have insomnia compared to patients without insomnia during a two-week period of active treatment under the conditions of routine clinical practice. *Materials and Methods*: The study population consisted of two groups of subjects with chronic back pain, a group with insomnia (“case”) with a total of 38 subjects and a control group without insomnia (“control”) with a total of 41 subjects, who filled out a set of measurement questionnaires: the McGill Pain Questionnaire and its short form (SF-MPQ), the Insomnia Severity Index (ISI) and the European Quality of Life Questionnaire of Life (Euro Qol; EQ-5D). Determination of the biomarkers of structural damage to the nervous tissue, neurofilament polypeptide (NEF—neurofilament polypeptide), neuron-specific enolase (NSE—neuron-specific enolase) and protein S100B was performed by measuring their concentrations in the blood using the ELISA method (enzyme immunosorbent assay). Statistical analysis of the collected data included a descriptive analysis, hypothesis testing methods and univariable and multivariable regression models. *Results*: At the end of the treatment visits, the level of pain remained higher in some subjects of the experimental group, but the statistical significance of the baseline difference disappeared because of the higher relative treatment response in the controls. Measured with a visual analogue scale, the treatment improved the patients’ quality of life much more in experimental than control subjects, as is proven by the statistically significant difference for the percent change from baseline (~31% vs. ~14%). At baseline, all three neurotropic biomarkers had significantly higher serum values in the subjects of the experimental group than in the control patients, which suggested more damage to the neuronal structures. During the treatment course, their serum concentrations decreased, from 36% to 95%, but for S100B, unlike NES and NEF, there was no statistically significant difference between the study groups at the end of the treatment visits. *Conclusions*: The results of the study have immediate scientific and practical significance because they contribute to new knowledge about the place and role of insomnia in patients with cervical and/or lumbar pain who are treated with physical medicine methods in the conditions of routine clinical practice. The treatment of insomnia should be an indispensable part of therapeutic treatment for patients with back pain.

## 1. Introduction

Insomnia is one of the most frequently reported comorbidities in chronic spine pain [1]. Studies show that people with chronic back pain are 18 times more likely to experience insomnia compared to people without chronic back pain [2]. If untreated, insomnia negatively affects physical symptoms, mood, sensitivity to pain, fatigue and health-related quality of life [3]. It is associated with lower productivity and increased absenteeism [4]. All of the above represents a serious public health problem that is rarely solved by treatment [5]. However, the underlying mechanisms that explain the relationship between sleep and pain are still not fully understood. Many studies talk about the connection between sleep and chronic pain in the spine, that is, that different sleep disorders and insomnia are correlated with the intensity of pain in patients with chronic pain in the spine. The complex processes of sleep and chronic pain seem to have an overlapping mechanism, which may explain their often-established bidirectional relationship [6]. Also, people with chronic back pain with high levels of pain intensity, comorbid musculoskeletal pain and anxiety and/or depression are more likely to have insomnia.

Evidence strongly suggests a bidirectional relationship, with pain and sleep coexisting and influencing each other [7]. There is evidence that insomnia and pain share similar pathways, such as the mesolimbic dopaminergic pathways and serotoninergic pathways. In general, pain is associated with an increased stress response and increased arousal, which can negatively affect sleep [8]. In addition, limited sleep loss has a deactivating effect on several analgesic systems, while activating hyperalgesic systems [8]. Moreover, sleep impairment can lead to low-grade inflammatory responses [9], which have been confirmed to potentially affect brain function and increase pain sensitivity [10]. This two-way relationship creates a vicious cycle—increased pain that interferes with sleep and sleep disturbance that worsens the pain.

One of the research methods can be monitoring the dynamics of appropriate biomarkers of the morphological and functional integrity of the nervous system. So, for example, it was demonstrated that sleep deprivation in humans significantly increases morning serum concentrations of NSE and S100B by about 20% and selectively compared to some other biomarkers of nervous tissue, such as amyloid peptides beta 1–40 and 1–42 [11]. Patients with insomnia (chronic insomnia disorder—CID) have elevated serum levels of NEF, NSE and S100B, which indicates the existence of a damaged microstructure of nervous tissues, including neurons, astrocytes and synapses, which are involved in the neuronal mechanisms of circadian rhythm homeostasis [12,13]. On the other hand, data on the mentioned biomarkers of structural damage to the nerve tissue in patients with cervical and/or lumbar pain are very rare. In one study, the immunoreactivity for neurofilaments (NF200) and neuropeptides such as substance P (SP) and vasoactive intestinal peptide (VIP) in the fibers innervating the intervertebral lumbar discs was observed to be significantly more intense in subjects with back pain than in healthy subjects [14]. Other studies indicate that NEF, NSE and S100B are among the biomarkers of structural damage to the neural tissue in spinal cord injuries [15].

Since insomnia has been shown to worsen pain, mood and physical functioning, it can negatively affect not only the quality of life, but also the clinical outcomes of patients with chronic cervical and lumbar pain, and insomnia should be treated as an indispensable part of pain management in patients with cervical and lumbar pain [11].

In this context, the primary objective of the study is to determine whether there is a difference in physical therapy outcomes (Pain Intensity) in patients with cervical and/or lumbar pain who have insomnia compared to patients without insomnia during a two-week period of active treatment under the conditions of routine clinical practice.

The secondary objectives of the research are as follows:

1. To determine the significance of the difference in the change in quality of life during physical therapy in patients with cervical and/or lumbar pain who simultaneously have insomnia, compared to patients without clinically significant insomnia, during a two-week period of active treatment under the conditions of routine clinical practice.
2. To examine the significance of the differences in the serum concentrations of biomarkers of the morphological and functional status of the nerve tissue, neurofilament polypeptide (NEF—neurofilament polypeptide), neuron-specific enolase (NSE—neuron-specific enolase) and protein S100B in patients with cervical and/or lumbar pain who simultaneously have insomnia compared to patients without clinically significant insomnia during a two-week period of active treatment under the conditions of routine clinical practice.

## 2. Materials and Methods

### 2.1. Research Design

This research is an open, retrospective and prospective clinical study with the follow-up of subjects and collection of study data according to a case-control design, in the conditions of routine clinical practice, in patients with cervical and/or lumbar pain lasting at least four weeks, for whom physical therapy is indicated, and who either have or do not have clinically significant insomnia before starting physical therapy. Research was conducted in the Department of Physical Medicine and Rehabilitation of the University Clinical Center Kragujevac in Kragujevac, Serbia during 2021, 2022 and 2023. The study was conducted after obtaining the approval of the competent Ethics Committee.

### 2.2. Study Population

The study population consisted of two groups of subjects, a group with insomnia (“case”) and a control group without insomnia (“control”), who were included in the study after signing an informed consent with previous full information. The successive inclusion of respondents during the duration of the study, according to the criteria for inclusion and exclusion and according to the number determined by the calculation of the study sample, resulted in a total number of 79 respondents (38 in the experimental group and 41 in the control group).

The evaluation of cervical and lumbar pain and the prescription of physical therapy were carried out by a doctor specializing in physical medicine and rehabilitation, according to a valid, well-founded methodological approach. All subjects included in the study were additionally referred to a psychiatrist for the diagnostic evaluation of the insomnia and the determination of appropriate therapy. Three study visits were planned: initial (before the start of therapy), after 7 days and after 14 days during physical therapy. Active evaluation of the study outcomes was performed during the first and last visit, which corresponded to the period of active physiotherapy treatment.

The inclusion criteria were as follows: persons aged 18 to 65 of both sexes, the presence of cervical and/or lumbar pain lasting at least four weeks requiring treatment with physical therapy methods, and voluntary participation of the subjects in the study. The special criteria for inclusion in the study group of subjects with insomnia were the values of the Insomnia Severity Index—ISI (Insomnia Severity Index) score ≥ 15.

The exclusion criteria were as follows: persons younger than 18 years and older than 65 years, the existence of an unstable chronic disease (except for pain syndrome), refusal of the subject to participate in the study, pregnancy or breastfeeding (for female subjects) and the existence of any disease, condition or circumstances that, in the judgment of the researcher, interfered with the subject’s participation in the study and adherence to the study procedures.

The subject could be excluded from the study at any time according to the individual clinical judgment of the researcher, the decision of the subject himself or the request of the competent regulatory bodies.

### 2.3. Research Instruments

The McGill Pain Questionnaire, Short Form (SF-MPQ) [16], is a questionnaire that assesses multiple aspects of pain and is considered a multidimensional measure of pain quality. Three dimensions of pain are examined: sensory–discriminative, affective–motivational and cognitive–evaluative. It consists of groups of words that describe the pain, but also gives numerical indexes to the different dimensions of pain. The McGill Questionnaire consists of three subscales:

(1) VRS (Verbal Response Scale): consists of 15 representative words that describe the type of pain. The patient ranks each on a scale from 0 to 3, where 0 means “I felt no pain”, 1 “I felt a little pain”, 2 “I felt moderate pain” and 3 “I felt a lot of pain”.
(2) PPI (Present Pain Intensity): PPI is a scale of current pain intensity. The patient indicates the current pain intensity on a scale of 0 to 5.
(3) VAS (Visual Analogue Scale): a one-dimensional pain scale.

The Insomnia Severity Index (ISI) [17] is a seven-question questionnaire that is given to the respondent to assess recent problems with falling asleep, maintaining sleep, early awakening and the consequences of insomnia, using five-level points (from 0—none to 4—very, a lot). The ISI score is obtained by summing the points for each item, and it ranges from 0 to 28. Higher scores correspond to insomnia of greater intensity, and can be classified into four categories: no insomnia (0–7), borderline insomnia (8–14), clinically clear insomnia—moderately severe (15–21) and clinically clear insomnia—severe (22–28).

The European Quality of Life (EuroQol; EQ-5D) [18] a general standardized indicator of quality of life that assesses five areas: mobility, self-care, daily activities, pain/discomfort and mood.

A designed questionnaire on the basic socio-demographic characteristics of the respondents and the characteristics of the disease (type of pain syndrome, pharmacotherapy, presence of other diseases, etc.) was used to collect data on other research variables.

The determination of the biomarkers of structural damage to the nervous tissue, neurofilament polypeptide (NEF—neurofilament polypeptide), neuron-specific enolase (NSE—neuron-specific enolase) and protein S100B, was performed by measuring their concentrations in blood samples taken from the subjects at the beginning and at the end of physical therapy. Samples were collected in the mornings and centrifuged for 5 min at 2000 rpm to separate the serum. A quantity of 3 mL of serum from each patient was frozen at −80 °C until analysis. The serum protein concentrations of NEF, NSE and S100B were measured in triplicate by the ELISA method (enzyme-linked immunosorbent assay) in accordance with the manufacturer’s instructions.

### 2.4. Research Variables

The primary dependent variable was the numerical score of the short form of the McGill Pain Questionnaire (SF-MPQ), while the primary independent variable was clinically significant insomnia, expressed by an ISI score ≥ 15.

The secondary dependent variables were the scores of the individual domains of the SF-MPQ questionnaire, the quality of life expressed by the numerical value of the score of the EQ-5D scale and the values of its individual domains, and the values of the serum protein concentrations of NEF, NSE and S100B.

The confounding variables were the sex, age, associated somatic comorbidities and mental disorders, health habits and lifestyle (e.g., consumption of cigarettes, alcohol and coffee) and socio-economic characteristics of the subjects of interest for the study outcomes (e.g., education, socio-economic status).

### 2.5. Power of Study and Sample Size

The sample calculation was based on the results of previous studies in which the effects of various physical therapy modalities were assessed using the McGill Short Pain Assessment Questionnaire (SF-MPQ), including studies on patients with low back pain [1,13]. The change in the descriptive score (the sum of the sensory and affective–emotional domain scores) at the end of the study compared to the beginning of treatment (T-PRI, total pain rating index) was considered as the primary dependent variable. Accordingly, it was estimated that in the control group, the value of the T-PRI score would decrease by 55% with a standard deviation of 22.5%, and that in the “case” group this decrease would be at least 15% greater than in the control group. Taking into account the mentioned parameters, the alpha error values of 0.05, the power of the study, 0.8, the ratio of the number of subjects in the groups, 1:1, and the *t*-tests for the two independent samples, with two-sided comparison using the appropriate program, the number of 36 subjects in each group was calculated. The total study sample was rounded up to 80 subjects, with 40 in each study group. By the successive inclusion of subjects according to the inclusion and exclusion criteria, we obtained a sample of 38 in the experimental group and 41 in the control group.

### 2.6. Statistical Data Processing

Statistical analysis of the collected data included a descriptive analysis and univariable and multivariable regression models. The analysis of the numerical data included measures of central tendency and variability, Student’s *t*-test or Wilcoxon Mann–Whitney test (depending on the type of distribution) and linear regression. The categorical variables were described descriptively, and the differences in frequency between groups were tested by the chi-square test and binary logistic regression. The basic statistical model for the analysis of the primary outcome, the change in score on the McGill questionnaire, was univariate and multivariate linear regression. The threshold probability values for all tests were set at *p* ≤ 0.05.

## 3. Results

The study population included a total of 79 patients, 38 in the experimental group and 41 in the control group. In general, there were no significant differences in the patients’ basic demographic characteristics between study groups (Table 1). In average, the patients were in their middle-fifth decade of life, and the majority were women, employed, married, living with their partners within urban settings and with sporadic coffee consumption. A substantial number of the study subjects were smokers and had somatic diseases, for which they used appropriate pharmacotherapies. The pain lasted about 4.5 months before the study inclusion, and it was treated primarily with various combinations of medical approaches, mainly with a plethora of different analgesic medicines. Night pain troubled many study subjects, too.

At baseline, the patients with insomnia had significantly higher levels of pain and a significantly lower quality of life than the subjects in the control group, as analysis of the scores of the clinical rating scales showed (Table 2). The insomnia itself was much worse in patients of the experimental group than in subjects of the control group, because its presence was an inclusion criterion for the between-group patients’ allocation (risk factor exposure and non-exposure).

At the end of the treatment visits, in the patients of both study groups, a significant treatment response appeared in almost all research domains. In the control study subjects, there were significant differences in the values of SF-MPQ VRS 2 in comparison with SF-MPQ VRS 1 (*p* < 0.001), SF-MPQ VAS2 in comparison with SF-MPQ VAS1 (*p* < 0.001), SF-MPQ PPI2 in comparison with SF-MPQ PPI1 *p* < 0.001), QALY2 in comparison with QALY1 (*p* < 0.001) and EQ5D VAS2 in comparison with EQ5D VAS1(*p* < 0.001). In the subjects of the experimental group, there were significant differences in the values of SF-MPQ VRS 2 in comparison with SF-MPQ VRS 1 (*p* < 0.001), SF-MPQ VAS2 in comparison with SF-MPQ VAS1 (*p* < 0.001), SF-MPQ PPI2 in comparison with SF-MPQ PPI1 (*p* < 0.001), QALY2 in comparison with QALY1 (*p* < 0.001) and EQ5D VAS2 in comparison with EQ5D VAS1 (*p* < 0.001).

At the end of the treatment visits, the level of pain remained higher in some subjects of the experimental group, but the statistical significance of the baseline difference disappeared because of the higher relative treatment response in the controls (measured by the percent change from baseline). On the other hand, the treatment response for the quality of life domains showed different intensities for the separate measurement outcomes—utility score and visual analog scale.

Measured with the visual analogue scale, the treatment improved the patients’ quality of life much more in the experimental subjects than the control subjects, as is proven by the statistically significant difference in the percent change from baseline (~31% vs. ~14%). However, the utility scores of the patients in both study groups improved similarly, by ~11–12% in the percent change from baseline and by a median of ~9–10 points in the absolute value change.

As expected, the insomnia improved much more in patients of the experimental group than in subjects of the control group. In the control patients, the values of ISIT2 and ISIT1 did not differ significantly from each other (*p* = 0.134). In contrast, in the patients with clinically important insomnia, the values of ISIT2 and ISIT1 differed significantly from each other (*p* < 0.001). Consequently, both the percent change from baseline and the absolute change in value of the patients’ Insomnia Severity Index total scores differed significantly between the study groups, showing much improvement in the subjects with clinically important insomnia at baseline.

At baseline, all three neurotropic biomarkers had significantly higher serum values in the subjects of the experimental group than in the control patients, which suggested more damage to the neuronal structures (Table 3). During the treatment course, their serum concentrations decreased by 36% to 95%, but for S100B, unlike NES and NEF, there was no statistically significant difference between the study groups at the end of the treatment visits. In addition, there were no significant differences in the values of S100B2 in comparison to S100B1, neither in the control study subjects (*p* = 0.236) nor in the patients with pain and clinically important insomnia (*p* = 0.215).

On the other side, in the control study subjects there were significant differences in the values of NSE2 in comparison with NSE1 (*p* < 0.001) and the values of NEF2 in comparison with NEF1 (*p* = 0.001). The same was true for the patients in the experimental group for the values of NSE2 in comparison with NSE1 (*p* < 0.001) and for the values of NEF2 in comparison with NEF1 (*p* = 0.001).

Table 4 shows the robust regression, univariable and multivariable models, predictors that had a significant impact on treatment outcomes expressed through the SF-MPQ VRS differences and the SF-MPQ VAS differences. We chose the absolute change values of the SF-MPQ scores for the regression outcome, instead of the percent change from baseline, based on the statistical stability (performance) of the two final, multivariable models. A significant, independent predictor for improvement in the SF-MPQ VRS scores was the use of psychotropic drugs (adjusted b = 3.640, *p* = 0.049), but neither the improvement in the ISI total score (adjusted b = 0.130, 0.381) nor the decrease of the serum concentrations of NSE (adjusted b = 0.083, *p* = 0.144) were significant predictors. Taking into account the SF-MPQ VAS scores, the presence of married marital status showed significant prediction for unfavorable outcomes (adjusted b = −0.012, *p* = 0.017), the decrease in the serum concentrations of NEF was a significant predictor of good treatment responses (adjusted b = 0.001, *p* = 0.021), and the decrease in the serum concentrations of NSE did not significantly predict the therapeutic outcome (adjusted b = 0.019, *p* = 0.233).

## 4. Discussion

The prevalence of insomnia among chronic pain patients is greater in comparison with the general population. When the clinical history indicates a straightforward diagnosis of chronic pain syndrome, patients will complain of insomnia as part of their symptomatology. It is imperative to manage their underlying illness to alleviate their sleep disorder.

Chronic back pain, with high rates of disability and socio-economic costs, is a public health problem of great concern worldwide. Research has shown that patients with chronic pain in the spine have certain limitations in performing daily activities, work incapacity, disability and consequently reduced quality of life [19,20]. Also, regular treatment and limited working ability impose a large financial burden on patients, which additionally contributes to a significant psychological burden [21].

The multidimensional negative impact of chronic back pain results in poorer quality of life in patients with chronic pain compared to the general population and patients with other long-term conditions. Compared to the general population and patients with long-term conditions, patients with chronic back pain had significantly lower mean values of quality of life in all domains, and the significant ones identified were impairments in physical functioning, impairments in professional life, impairments in relationships and family life, social life, sleep disturbance and mood disturbance [22,23].

Insomnia has long been known to be related to chronic pain conditions. Insomnia is caused by reduced sleep quality and duration, a greater amount of time to fall asleep, poor daytime function, and greater sleep dissatisfaction and distress. Because insomnia has been shown to worsen pain, mood and physical functioning, it could negatively impact the clinical outcomes of patients. Understanding these factors will help clinicians educate patients and will facilitate the formulation of more effective treatment plans. However, many clinics have insufficient resources or expertise that provide a detailed sleep assessment for patients who are complaining of insomnia [24,25].

The prevalence of insomnia among chronic pain patients is greater in comparison with the general population. When the clinical history indicates a straightforward diagnosis of chronic pain syndrome, patients will complain of insomnia as part of their symptomatology [26,27,28].

The complex processes of sleep and chronic pain appear to have an overlapping mechanism, which may explain their often-established bidirectional relationship. Many studies talk about the connection between sleep and chronic pain in the spine, that is, that different sleep disorders and insomnia are correlated with the intensity of pain in patients with chronic pain in the spine. The connection between sleep and chronic pain in the spine is confirmed by numerous studies [5,29,30].

Our results also confirm the results of many studies that have shown that in people with cervical and lumbar pain who also have insomnia, there is an elevated pain scale.

Such a condition not only disrupts sleep, but also disables a person in everyday activities and consequently leads to a poor quality of life, which suggests the importance of solving this problem. Also, disturbed sleep can reduce the effectiveness of pain management and delay recovery [31]. Research that assessed the impact of insomnia and daytime sleepiness on the quality of life of patients suffering from chronic back pain showed that rooms that suffered from insomnia and were more sleepy during the day were characterized by a lower perception of quality of life, suggesting that insomnia is an important predictor affecting the quality of life in people with chronic back pain [19]. Our results also support this. Bearing in mind that after the treatment of insomnia and physiatry treatment, the quality of life improved in our experimental group of subjects, we confirm that the treatment of insomnia should be an indispensable part of the therapeutic treatment for patients with back pain.

Also, is not sufficiently known whether and what kind of connection exists between possible changes in the serum concentrations of the mentioned nervous tissue proteins, the condition caused by cervical and/or lumbar pain syndrome and insomnia. Studies that have dealt with these relationships are rare [32]. If such a connection were significant, this knowledge would indicate that in persons with neck and back pain who also have chronic insomnia, there is significant structural damage to the nervous tissue, either at the level of the peripheral nerves or in the central nervous system, including the spinal cord and structures that are involved in the central regulation of the sleep–wake cycle. An additional unknown concerns the therapeutic possibilities in the presence of such damage, and pharmacotherapy and physical therapy methods could be of importance in this regard [33]. New knowledge about the connection between pain and insomnia in the practice of physical medicine and rehabilitation would contribute to the theoretical base of further research in this area, including efforts to better understand the importance of prevention and therapy in the field of improving the mental health of patients with pain syndromes.

In this context, our research on the differences in the serum concentrations of the biomarkers of morphological and functional status of the nerve tissue, neurofilament polypeptide (NEF—neurofilament polypeptide), neuron-specific enolase (NSE—neuron-specific enolase) and protein S100B in patients with cervical and/or lumbar pain who simultaneously have insomnia compared to patients without clinically significant insomnia during a two-week period of active treatment under the conditions of routine clinical practice makes a significant contribution.

After the treatment of insomnia in our experimental group of subjects, there was a significant improvement in sleep. It was also noted that the quality of life improved significantly more than in the control subjects. Although before the treatment of insomnia all three neurotropic biomarkers had significantly higher serum values in the subjects of the experimental group than in the control patients, which indicates greater damage to the neuronal structures in the comorbid patients, during treatment, their serum concentrations decreased. Thus, the positive effects of treating insomnia in patients with cervical and lumbar pain with the simultaneous application of physiatry treatment have been demonstrated. Such findings suggest the importance of additional research in the future.

## 5. Conclusions

Neurotropic biomarkers had significantly higher serum values in subjects of the experimental group with insomnia than in the control group, which indicates greater damage to neuronal structures in subjects with insomnia. During treatment, their serum concentrations decreased. At the end of the treatment visits, the patients in both study groups showed a significant response to treatment in almost all research domains. Bearing in mind that after the treatment of insomnia and physiatry treatment, the quality of life improved in our experimental group of subjects, we confirm that the treatment of insomnia should be an indispensable part of the therapeutic treatment for patients with back pain.

## Figures and Tables

**Table 1:** The demographic characteristics of the patients in the study.

Variable	All Patients (*n* = 79)	Experimental Group-1 (*n* = 38)	Control Group-2 (*n* = 41)	*p*
Age (years), mean ± sd	46.9 ± 11.3	48.2 ± 12.4	45.6 ± 10.2	*p* = 0.304
Gender (female), *n* (%)	55 (69.6)	29 (76.3)	26 (63.4)	*p* = 0.213
Work, *n* (%)				
Employed	48 (60.8)	24 (63.2)	24 (58.5)	*p* = 0.972
Retired	9 (11.4)	4 (10.5)	5 (12.2)	
Unemployed	20 (25.3)	9 (23.7)	11 (26.8)	
Other	2 (2.5)	1 (2.6)	1 (2.4)	
Working time (years), median (range)	15 (1–41)	14 (1–41)	15 (1–40)	*p* = 0.411
Night work (yes)	8 (15.7)	4 (16.0)	4 (15.4)	*p* = 1.000
Marital status				
Single	14 (17.7)	5 (13.2)	9 (22.0)	*p* = 0.633
Married	54 (68.4)	27 (71.1)	27 (65.9)	
Divorced	8 (10.1)	5 (13.2)	3 (7.3)	
Widowed	3 (3.8)	1 (2.6)	2 (4.9)	
Housing				
Alone	7 (8.9)	4 (10.5)	3 (7.3)	*p* = 0.580
Partnership	47 (59.5)	25 (65.8)	22 (53.7)	
Children	8 (10.1)	2 (5.3)	6 (14.6)	
Parents	4 (5.1)	2 (5.3)	2 (4.9)	
Other	13 (16.5)	5 (13.2)	8 (19.5)	
Residence				
Urban	41 (51.9)	18 (47.4)	23 (56.1)	*p* = 0.208
Rural	14 (17.7)	5 (13.2)	9 (22.0)	
Suburban	24 (30.4)	15 (39.5)	9 (22.0)	
Somatic disease (yes)	33 (41.8)	17 (44.7)	16 (39.0)	*p* = 0.607
Somatic drugs (yes)	31 (39.2)	17 (44.7)	14 (34.1)	*p* = 0.335
Mental disease (yes)	4 (5.1)	3 (7.9)	1 (2.4)	*p* = 0.347
Psychotropics (yes)	30 (38.0)	15 (39.5)	15 (36.6)	*p* = 0.792
Pain duration (days)	90 (3–730)	90 (8–730)	90 (3–730)	*p* = 0.992
Pain treatment				
Combination	40 (50.6)	19 (50.0)	21 (51.2)	*p* = 0.775
Drugs, only	10 (12.7)	6 (15.8)	4 (9.8)	
No	29 (36.7)	13 (34.2)	16 (39.0)	
Night pain (yes)	63 (79.7)	33 (86.8)	30 (73.2)	*p* = 0.131
Analgesic use (yes)	67 (84.8)	34 (89.5)	33 (80.5)	*p* = 0.266
Smoking (yes)	34 (43.0)	19 (50.0)	15 (36.9)	*p* = 0.229
Coffee use (daily)				
>4 cups	10 (12.7)	4 (10.5)	6 (14.6)	*p* = 0.374
1–3 cups	59 (74.7)	28 (73.7)	31 (75.6)	
No	10 (12.7)	6 (15.8)	4 (9.8)	
Alcohol use				
Every day	0 (0)	0 (0)	0 (0)	*p* = 0.206
Sporadically	15 (19.0)	5 (13.2)	10 (24.4)	
No	64 (91.0)	33 (86.8)	31 (75.6)	
Energy drink use				
Every day	1 (1.3)	1 (2.6)	0 (0)	*p* = 0.561
Sporadically	7 (8.9)	2 (5.3)	5 (12.2)	
No	71 (89.9)	35 (92.1)	36 (87.8)	

**Table 2:** Clinical rating scale scores, at baseline (1) and at end of treatment visits (2), with their absolute change from baseline (DA—difference, absolute) and percent change from baseline (DP—difference, percent).

Variable	All Patients	Experimental Group	Control Group	*p*
ISI1	11 (0–24)	17.0 (15–24)	4 (0–11)	*p* < 0.001 *
ISI2	4 (0–21)	6.5 (0–21)	3 (0–13)	*p* = 0.004 *
ISI DA	−3 (−22–5)	−10.5 (−22–4)	−0.1 (−7–5)	*p* < 0.001 *
ISI DP	−50.0 (−100.0–2900.0)	−61.8 (−100.0–23.5)	−40.0 (−100.0–2900.0)	*p* = 0.036 *
SF-MPQ VRS 1	19.5 ± 8.0	17.7 ± 8.2	21.4 ± 7.3	*p* = 0.039 *
SF-MPQ VRS 2	9 (1–39)	9.51 ± 6.94	11.87 ± 8.19	*p* = 0.151
SF-MPQ VRSDA	−8.8 ± 7.9	−9.5 ± 8.8	−8.1 ± 7.1	*p* = 0.459
SF-MPQVRS DP	−50.0 (−97.1–130.8)	−52.2 (−88.9–41.2)	−50.0 (−97.1–130.8)	*p* = 0.728
SF-MPQVAS 1	59.4 ± 20.3	65.8 ± 19.6	53.4 ± 19.2	*p* = 0.006 *
SF-MPQVAS 2	31 (1–72)	37.16 ± 19.7	32.71 ± 19.08	*p* = 0.276
SF-MPQVAS DA	−24.5 ± 22.9	−28.7 ± 19.9	−20.7 ± 25.0	*p* = 0.124
SF-MPQVAS DP	−43.8 (−98.5–300.0)	−42.7 (−98.5–50.0)	−46.2 (−95.8–300.0)	*p* = 0.566
SF-MPQPPI1	2 (0–5)	2 (0–5)	2 (0–5)	*p* = 0.058
SF-MPQPPI2	1 (0–4)	1 (0–4)	1 (0–4)	*p* = 0.244
QALY1	0.78 (0.24–0.90)	0.72 (0.24–0.88)	0.78 (0.34–0.90)	*p* = 0.007 *
QALY2	0.82 (0.06–1.00)	0.80 (0.06–1.00)	0.85 (0.62–1.00)	*p* = 0.025 *
QALY DA	0.10 (−0.46, 0.59)	0.10 (−0.46, 0.59)	0.10 (−0.08, 0.50)	*p* = 0.914
QALY DP	11.1 (−86.0–228.2)	11.8 (−86.0–228.2)	11.1 (−9.7–143.9)	*p* = 0.906
EQ5D VAS1	59.8 ± 17.0	52.9 ± 15.4	66.1 ± 16.1	*p* < 0.001 *
EQ5D VAS2	74.7 ± 15.8	71.7 ± 16.1	77.5 ± 14.7	*p* = 0.098
EQ5D VAS DA	14.9 ± 18.8	18.8 ± 19.8	11.4 ± 17.4	*p* = 0.080
EQ5D VAS DP	21.4 (−33.3–1420.0)	30.6 (−33.3–1420.0)	14.3 (−33.3–300.0)	*p* = 0.015 *

All variables are numerical; due to small variation magnitude of SF-MPQ PPI values, their DA and PA analysis was omitted; *—significant difference.

**Table 3:** The serum neurotropic biomarker values of the patients during the study.

Variable	All Patients	Experimental Group	Control Group	*p*
S100B1	545.6 (1.6–5897.6)	661.6 (17.6–5897.6)	449.6 (1.6–3329.6)	*p* = 0.047 *
S100B2	464.0 (32.0–4608.0)	424.0 (32.0–4608.0)	480.0 (56.0–4432.0)	*p* = 0.645
S100BDA	134.4 (−5681.6–4158.0)	−187.6 (−5681.6–4158.0)	150.4 (−3273.6–3966.4)	*p* = 0.148
S100BDP	23.6 (−98.3–19,400)	−36.1 (−97.2–1763.6)	46.5 (−98.3–19,400)	*p* = 0.073
NSE1	10.6 (0.3–56.8)	14.1 (2.4–56.8)	8.8 (0.3–54.1)	*p* = 0.006 *
NSE2	0.76 (0.65–1.33)	0.74 (0.65–1.33)	0.83 (0.66–1.24)	*p* = 0.016 *
NSEDA	−9.7 (−55.9–0.9)	−13.5 [−55.9–(−1.6)]	−7.8 (−53.0–0.9)	*p* = 0.005
NSEDP	−91.5 (−98.8–255.6)	−95.1 [−98.8–(−67.6)]	−88.3 (−98.0–255.7)	*p* = 0.001 *
NEF1	3207.0 (16.6–19,390.2)	4738.5 (80.2–17,268.0)	1679.1 (16.6–19,390.2)	*p* = 0.002 *
NEF2	343.7 (6.4–8223.4)	369.2 (6.6–1960.7)	326.9 (6.4–8223.4)	*p* = 0.634
NEFHDA	−2459.1 (−19,089.4–6919.0)	−4161.1 (−16,242.3–73.5)	−809.0 (−19,089.4–6919.0)	*p* < 0.001 *
NEFHDP	−84.3 (−99.8–11,357.7)	−91.3 (−99.8–91.8)	−70.2 (−99.8–11,357.7)	*p* < 0.001 *

* statistical significance.

**Table 4:** Robust regression, univariable and multivariable model of predictors that had significant impact on treatment outcomes, expressed through SF-MPQ VRS difference and SF-MPQ VAS difference.

Variable	Univariable Model				Multivariable Model			
	SF-MPQ VRS.DA		SF-MPQ VAS.DA		SF-MPQ VRS.DA		SF-MPQ VAS.DA	
	b	*p*	b	*p*	b	*p*	b	*p*
Insomnia	1.450	0.398	7.189	0.167				
Female gender	0.118	0.945	2.819	0.617				
Age	0.087	0.323	0.061	0.814				
Working, employed	0.085	0.962	−0.024	0.997				
Working, night shifts	−1.285	0.678	1.495	0.859				
Marital status, married	−0.191	0.918	−11.758	0.029 *			−0.012	0.017
Urban housing	0.643	0.785	7.043	0.247				
Somatic disease	−2.213	0.205	−8.088	0.138				
Mental disease	1.334	0.774	0.865	0.954				
Psychotropic	4.014	0.026 *	−1.934	0.712	3.640	0.049		
Pain duration (days)	<0.001	0.598	−0.004	0.221				
Night pain at baseline	4.905	0.008	4.745	0.383				
Analgesics at baseline	−0.381	0.876	−1.956	0.812				
Smoking	0.662	0.702	7.265	0.173				
Coffee use (1–3 cups)	−2.128	0.423	−7.168	0.327				
Coffee use (>3 cups)	−0.500	0.870	1.191	0.919				
Alcohol use, sporadically	−2.371	0.217	−0.601	0.939				
Energy drink use	−0.642	0.715	−8.679	0.332				
QALY.DA	4.207	0.313	25.411	0.115				
EQVAS.DA	0.047	0.239	0.128	0.378				
ISIT.DA	0.302	0.047 *	0.746	0.112	0.130	0.381		
S100B.DA	<−0.001	0.150	−0.002	0.227				
NSE.DA	0.110	0.019 *	0.240	0.095 *	0.083	0.144	0.019	0.233
NEF.DA	<0.001	<0.001 **	<0.001	0.003 *			0.001	0.021

* Variables which were included in multivariable model; ** due to multicollinearity, the variable NEF.DA was not included in the multivariate model of SF-MPQ VRS.DA.

## Data Availability

The original contributions presented in the study are included in the article; further inquiries can be directed to the corresponding author.
